# Metal Ions in Biological Systems, Volume 32

**DOI:** 10.1155/MBD.1997.111

**Published:** 1997


### METAL IONS IN BIOLOGICAL SYSTEMS
### Volume 32
**Edited by A. Sigel and H. Sigel, Marcel Dekker, Inc. (1996)**
**848 pages, ISBN 0-8247-9549-0**

| | |
|---|---|
| Chapter 1 : | Phosphate-metal ion interactions of nucleotides and polynucleotides, by C. Klevickis and C. Grisham |
| Chapter 2 : | Sugar-metal ion interactions, by S. Yano and M. Otsuka |
| Chapter 3 : | Dichotomy of metal ion binding to N1 and N7 of purines, by R. B. Martin |
| Chapter 4 : | General conclusions from solid state studies of nucleotide-metal ion complexes, by K. Aoki |
| Chapter 5 : | Solution structures of nucleotide-metal ion complexes. Isomeric equilibria, by H. Sigel and B. Song |
| Chapter 6 : | Stacking interactions involving nucleotides and metal ion complexes, by O. Yamauchi, A. Odani, H. Masuda and H. Sigel |
| Chapter 7 : | Effect of metal ions on the hydrolytic reactions of nucleotides and their phosphoesters, by S. Kuusela and H. Lönnberg |
| Chapter 8 : | Metal complexes of sulfur-containing purine derivatives, by E. Dubler |
| Chapter 9 : | Mechanistic insight from kinetic studies on the interaction of model palladium(II) complexes with nucleic acid components, by T. Rau and R. van Eldik |
| Chapter 10 : | Platinum(II)-nucleobase interactions. A kinetic approach, by J. Arpalahti |
| Chapter 11 : | NMR studies of oligonucleotide-metal ion interactions, by E. Sletten and N. A. Froysten |
| Chapter 12 ; | Metal ion interactions with DNA: considerations on structure, stability, and effects from metal ion binding, by V. G. Bregadze |
| Chapter 13 : | Electron transfer reactions through the DNA double helix, by T. J. Meade |
| Chapter 14 : | Role of metal ions in ribozymes, by A. M. Pyle |
| Chapter 15 : | Ternary metal ion-nucleic acid base-protein complexes, by M. Sabat |
| Chapter 16 : | Metal-responsive gene regulation and the zinc-metalloregulatory model, by D. A. Suhy and T. V. O'Halloran |
| Chapter 17 : | Role of iron-sulfur proteins in gene regulation, by M. C. Kennedy |
| Chapter 18 : | Current status of structure-activity relationships of platinum anticancer drugs : activation of the trans geometry, by N. Farrell |
| Chapter 19 : | Cisplatin and derived anticancer drugs : mechanism and current status of DNA binding, by M. J. Bloemink and J. Reedijk |
| Chapter 20 : | Proteins that bind to and mediate the biological activity of platinum anticancer drug-DNA adducts, by J. P. Whitehead and S. J. Lippard |
| Chapter 21 : | Interactions of metallopharmaceuticals with DNA : M. J. Clarke and M. Stubbs |

The first two chapters discuss interactions of metal ions with the phosphate and the sugar residues of a nucleotide.
Chapter 1 focuses on the stability and the structure as well in the solid state as in solution of the phosphate complexes, the last two sub-chapters being dedicated to the catalysis by metal ions in nonenzymatic displacement reaction at phosphorus.
Chapter 2 describes the interactions of metal ions with simple sugars (lactose, galactose, ...) and with the sugar residues of various biologically important molecules studied by various techniques like ESR, CD, X-ray diffraction, NMR.
Chapters 3 to 6 are devoted to the interactions between metal ions and nucleobases, nucleotides and oligonucleotides.
Chapter 3 presents a new analytical method that provides quantitative estimation of the binding of metal ions at N1 versus N7 of purines.
Chapter 4 presents X-ray structures and discusses the influence of the metal ion on the binding with nucleotides.

Chapter 5 gives the stability constants for the nucleosides-5'-phosphate complexes of alkaline earth ions, as well as $Mn^{2+}$, $Co^{2+}$, $Ni^{2+}$, $Cu^{2+}$, $Zn^{2+}$, and $Cd^{2+}$ allowing to quantify the isomeric equilibria when the metal can form complexes of different structures with a given nucleotide.
Chapter 6 discusses of the intermolecular stack formation between nucleotides and some metal ions like kinetically inert Pt(II) complexes containing aromatics ligands, alkaline earth and 3d metal ions. Some general aspects of selectivity in biological systems are given.
Chapter 7 is a study of the influence of metal ions on the most prominent reactions that nucleotides can undergo in aqueous solution : the acid-catalyzed depurination of the 2'-deoxyribonucleoside units of DNA and the hydrolysis and isomerization of the internucleosidic phosphodiester bond of RNA. The main emphasis is on kinetic studies carried out with monomeric model compounds.
Chapter 8 describes the solid state structures of several metal ion complexes of thiopurines, with a review of all the structural data available.
Chapters 9 to 12 deal with interactions of nucleobases and oligonucleotides with metal ions from the mechanistic, kinetic, methodologic and structural points of view.
Chapter 9 studies the reactivity and the factors controlling the reactivty of Pd(II) complexes which are model compounds for the less reactive Pt(II) antitumoral agents.
Chapter 10 presents a brief summary of the aqueous chemistry of cis-platin and a kinetic HPLC study of the interaction of Pt(II) with nucleobases.
Chapter 11 introduces the NMR techniques available to study the interactions of metal ions with oligonucleotides, from the point of view of the binding sites and the effect on the duplex DNA structure.
Chapter 12 discusses the dynamic structure of the DNA as well as of the surrounding hydrodynamic layer consisting of water and mobile ions.
Chapter 13 summarize the role of DNA as a mediator of electron transfer.
Chapter 14 presents the results of studies published after 1992 on the role of metal ions in ribozymes.
The goal of chapter 15 is to characterise the metal ion - nucleic acid - protein ternary complexes, in the case where the metal ion is directly bonded to the nucleic acid bases.
Chapter 16 reviews the potential role of zinc in the signal transduction and gene regulation and discusses some testable models.
Chapter 17 describes the new and unexpected discovery of the regulation function of the iron-sulfur proteins and, in particular, of the Fe-S cluster. A number of proteins related to gene expression, known or presumed to contain Fe-S clusters, for which no known function has been established yet, are presented.
Chapters 18 and 19 deal about platinum anticancer drugs.
Chapter 18 summarizes all the studiesd devoted to the trans-platin complexes, in particular trans-$[PtCl_2(py)_2]$ and trans-$[PtCl_2(NH_3)(quinoline)]$.
Chapter 19 focuses on the synthesis of platinum complexes, on structure-activity relationships, kinetic and on structural aspects and toxicity and concludes by presenting the perspective of these complexes and of some new generation ones.
Chapter 20 first places the subject into some historical context and then delineates how high-mobility group (HMG)-domain proteins have become a focus of intense current interest in attempts to elucidate the molecular mechanism of action of cisplatin.
Chapter 21 describes all the non-platinated complexes with anticancer activity described before 1995 and tries to point out some new interactions of metal ions with nucleic acids that suggest new pathways in metallochemotherapeutics.

In conclusion, this book is very helpful for every researcher who works in the field of biochemistry, bioinorganic chemistry, organometallic chemistry, and especially for everyone who deals with or is interested in the interactions of metal ions with nucleic acids.

